# Pathway to Cryogen Free Production of Hyperpolarized Krypton-83 and Xenon-129

**DOI:** 10.1371/journal.pone.0049927

**Published:** 2012-11-27

**Authors:** Joseph S. Six, Theodore Hughes-Riley, Karl F. Stupic, Galina E. Pavlovskaya, Thomas Meersmann

**Affiliations:** University of Nottingham, School of Clinical Sciences, Sir Peter Mansfield Magnetic Resonance Centre, Nottingham, United Kingdom; Albert Einstein College of Medicine, United States of America

## Abstract

Hyperpolarized (hp) ^129^Xe and hp ^83^Kr for magnetic resonance imaging (MRI) are typically obtained through spin-exchange optical pumping (SEOP) in gas mixtures with dilute concentrations of the respective noble gas. The usage of dilute noble gases mixtures requires cryogenic gas separation after SEOP, a step that makes clinical and preclinical applications of hp ^129^Xe MRI cumbersome. For hp ^83^Kr MRI, cryogenic concentration is not practical due to depolarization that is caused by quadrupolar relaxation in the condensed phase. In this work, the concept of stopped flow SEOP with concentrated noble gas mixtures at low pressures was explored using a laser with 23.3 W of output power and 0.25 nm linewidth. For ^129^Xe SEOP without cryogenic separation, the highest obtained MR signal intensity from the hp xenon-nitrogen gas mixture was equivalent to that arising from 15.5±1.9% spin polarized ^129^Xe in pure xenon gas. The production rate of the hp gas mixture, measured at 298 K, was 1.8 cm^3^/min. For hp ^83^Kr, the equivalent of 4.4±0.5% spin polarization in pure krypton at a production rate of 2 cm^3^/min was produced. The general dependency of spin polarization upon gas pressure obtained in stopped flow SEOP is reported for various noble gas concentrations. Aspects of SEOP specific to the two noble gas isotopes are discussed and compared with current theoretical opinions. A non-linear pressure broadening of the Rb D_1_ transition was observed and taken into account for the qualitative description of the SEOP process.

## Introduction

Nuclear magnetic resonance imaging (MRI) of the respiratory system using hyperpolarized (hp) ^129^Xe is increasingly attracting attention for clinical [1], [2], [3], [4], [5], [6] and preclinical research [7], [8] despite the associated lower signal intensities compared to the more established hp ^3^He MRI [9], [10]. Hp ^129^Xe provides additional information due to its chemical shift and tissue solubility [11] and its attractiveness is further augmented by the limited availability of the ^3^He isotope [12], [13]. The isotope ^83^Kr possesses a nuclear electric quadrupole moment (eQ) that may enable hp ^83^Kr to be used as a surface sensitive contrast agent and biomarker [14], [15].

Both noble gas isotopes, ^129^Xe (nuclear spin *I = 1/2*) and ^83^Kr (*I = 9/2*), can be hyperpolarized through spin exchange optical pumping (SEOP) with alkali metal vapor [16], [17], [18], [19]. Alternatively, dynamic nuclear polarization (DNP) at 1.2 K temperature was reported recently that allows for at least 7% hp ^129^Xe production [20]. For SEOP, the noble gases are typically diluted in helium - nitrogen mixtures and, in the case of ^129^Xe, the hp xenon is subsequently separated from the other gasses by a freeze-thawing cycle using a cold trap at 77 K [5], [21], [22], [23]. This process is not viable for hp ^83^Kr because of its rapid quadrupolar relaxation in the frozen state [24], [25]. Although cryogenic separation of hp ^129^Xe is straightforward in a physics or chemistry laboratory with acceptable losses [23], [26], it would be desirable to eliminate cryogen usage to facilitate hp ^129^Xe MRI applications in typical clinical and pre-clinical settings.

A high noble gas concentration in the SEOP gas mixtures would reduce the need for gas separation and could open up the pathway for cryogen free hp noble gas MRI. Unfortunately, a high noble gas density, [NG], adversely affects the obtained noble gas spin polarization, P_NG_, because it reduces the alkali metal electron spin polarization in the SEOP process. The adverse effect of [NG] on P_NG_ is further exacerbated by the diminishing effect of [NG] upon the spin exchange rate, 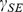 [21], [27], [28], [29], [30]. If cryogenic separation is omitted, a trade off between noble gas concentration and obtained spin polarization exists. For example, a spin polarization of approximately 1% was generated in a previously reported ^83^Kr SEOP experiments using a mixture of 95% krypton with 5% N_2_. Reducing the noble gas concentration to 25% krypton led to four fold higher spin polarization but the MR signal did not improve because polarization increase was offset by the noble gas dilution [31].

A potential solution for the conundrum to generate high *P*_NG_ at high noble gas concentrations is to reduce [NG] through decreasing the total pressure of the gas mixture containing a high percentage of the respective noble gas. Optical pumping far below ambient pressure had been the method of choice in many of the pioneering SEOP studies [16], [17], [32], [33], [34], but low pressure SEOP was largely abandoned with the advent of high power solid state lasers that provide better polarization at elevated gas pressures due to pressure broadening of the rubidium D_1_ transition. However, line narrowed high power diode array lasers have become available [28], [34], [35] that make pressure broadening less beneficial. Even non-narrowed (typically 2 nm linewidth) solid state lasers benefit from ^129^Xe SEOP at a gas pressure below ambient, as previously demonstrated by Imai et al. [36]. Unfortunately high spin polarization >12% was obtained (at 15 kPa pressure) only for mixtures with low xenon concentration leaving cryogenic separation as a remaining desirable step. However, the work by Imai et al. also demonstrated that recompression of hp ^129^Xe to ambient pressure after SEOP is feasible without significant losses in spin polarization. Recompression of the hp noble gas to ambient pressure would be a crucial step for intended low pressure SEOP usage for *in vivo* MRI applications.

In this work, ‘stopped flow’ (batch mode) SEOP [17] was utilized. In contrast to ‘continuous flow’ SEOP [5], [21], [22], [23], [37], [38], [39], [40] that is technically more demanding [22], [23], [40], ‘stopped flow’ SEOP is applied to a stagnant gas mixture until the steady state polarization has been reached. The hp noble gas is then shuttled through pressure equalization into a pre-evacuated chamber for high field MR detection without re-pressurization. The advantage of ‘stopped flow’ ^129^Xe SEOP was noted previously [41] and remarkably high ^129^Xe spin polarization were reported recently [28]. With the noticeable exception of the work by Fujiwara and coworkers [42], [43], pulmonary MRI typically uses hp gas in batched volumes. Therefore stopped flow SEOP may be of interest for pulmonary hp ^129^Xe MRI applications, in particular if it provides some advantages beyond current continuous flow methods.

To date, stopped flow SEOP is the only viable technique for hyperpolarizing noble gases with nuclear electric quadrupolar moment such as ^83^Kr [44], [45]. In this publication, stopped-flow SEOP was studied with mixtures containing 5–78% of either krypton or xenon at total gas pressures ranging from 5 kPa to 200 kPa and above. Current theory was applied to attempt a qualitative interpretation of the experimental data.

## Experimental

### 2.1. Stopped Flow SEOP

**Figure 1:**
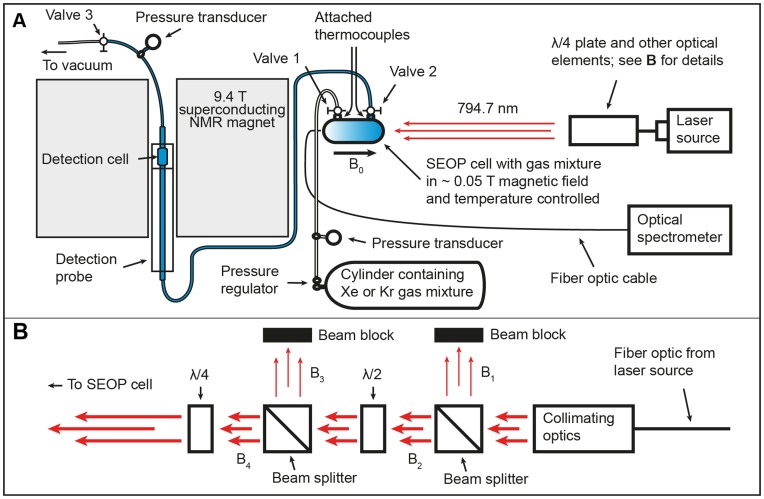
The experimental setup used for stopped flow SEOP. **A**. Shuttling to high-field detection. The hp mixture is transferred to the detection cell by pressure equalization after the noble gas mixtures are hyperpolarized in the SEOP cell for a time period of t_d_ by the stopped flow SEOP method. **B**. Outline of the optical elements used in Fig. 1A. The elements λ/2 plate and second beam splitter were used to control the laser irradiation (B_4_) to the SEOP cell (i.e. adjustment of the B_4_/B_3_ ratio - for details of power dependent measurements see *section 2.2*).

The experimental setup is sketched in Fig. 1. Mixtures containing various concentrations of ^129^Xe and ^83^Kr were hyperpolarized in borosilicate glass SEOP cells (length = 120 mm, inner diameter = 28 mm) containing ∼1 g Rb (99.75%; Alfa Aesar, Heysham, England, UK). The SEOP cell was housed in an aluminum oven with quartz windows and temperature controlled using heated air. The fringe field of a 9.4 T superconducting magnet provided the magnetic field of 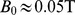 for the SEOP process. Unless otherwise specified, a line narrowed diode-array laser system (30 W, 0.25 nm linewidth Comet Module, Spectral Physics, Santa Clara, CA, USA) tuned to the D_1_ transition of Rb (794.7 nm) was used to irradiate the SEOP cell with collimated, circularly polarized light of 23.3 W power (incident at SEOP cell).

Steady state, nuclear spin polarization was reached after 6 minutes for ^129^Xe SEOP and after approximately 18 minutes for ^83^Kr SEOP. However, due to time restraints ^83^Kr SEOP times of only 8 minutes were used resulting to 80% completion of the built up, as verified by measurements at both high and low SEOP pressure. During SEOP, the gas mixture was contained within the SEOP cell with valve 2 closed (see Fig. 1A). Valve 1 was kept open initially to allow for pressure monitoring but was closed approximately 2 minutes before delivery. The borosilicate detection cell and PFA transfer tubing were evacuated (valve 3 open) during the SEOP duration. After SEOP completion, valve 3 was closed and valve 2 opened. Pressure equalization caused rapid hp gas transfer via 1.5 mm (inner diameter) PFA tubing into the 15 mm borosilicate detection cell. The detection cell located within a 9.4 T superconducting magnet and a Magritek Kea 2 spectrometer (Wellington, NZ) with custom-built probes tuned to the resonance frequencies of ^129^Xe (110.5 MHz) and ^83^Kr (15.4 MHz) where used for detection.

### 2.2. Laser Power Adjustment and Optical Measurements

In addition to the line narrowed Comet laser, two broadband 30 W Coherent (Santa Clara, CA, USA) fiber array packaged (FAP) lasers were also used as a non-narrowed laser system (2 nm linewidth) for SEOP efficiency comparison with the line narrowed Comet laser. Due to the experimental setting only 15.6 W of FAP laser power was used to irradiate the SEOP cell. To have a proper comparison between the narrowed and broadband laser systems the laser power of the narrowed laser was reduced to approximately match the power of the broadband system. Fig. 1B displays the optical elements used to reduce the power of the Comet laser. The first beam splitter in the path of the laser light was present in all experiments in this work and ensured that only a single plane of linearly polarized light would continue toward the SEOP cell. It was found that B_2_ = 19 B_1_ for the highly linear polarized Comet system and B_2_ = B_1_ for the FAP system (i.e. no linear polarization remaining due to passage through the long fibre optic cable of the FAP system). Laser power control was obtained through a λ/2 wave plate followed by a second beam splitter. By rotating the λ/2 wave plate the laser rejection (B_3_) was controlled, thus enabling the power control for the laser irradiation (B_4_) of the SEOP cell without changes in the irradiation profile (i.e. wavelength and spatial distribution). The incident laser power was measured at the SEOP cell using a Coherent PM150-50C water-cooled power meter. The same power adjustment procedure was also used for the power dependent measurements described in *section 4.9*.

The rubidium absorption linewidth in the presence of pure krypton, xenon, N_2_, and a Xe - N_2_ mixture was measured through absorption experiments similar to those by Driehuys and co-workers [46]. An incandescent light source with a consistent emission over the observed wavelengths irradiated the SEOP cell in place of the laser. A fibre optic cable leading to the optical spectrometer, HR2000+ Ocean Optics (Dunedin, Fl, USA) with a spectral resolution of 0.04 nm was placed at the rear of the SEOP cell to measure the D_1_ absorption line width at 794.72–795.15 nm.

### 2.3. Temperature Control

The temperature of the SEOP cell inside the oven was maintained by an inflow of heated air near the back of the cell. Two thermocouples attached to the SEOP cell were used to measure the cell temperature. The first thermocouple was placed at the frontal region of the cell (i.e. in approximately 10 mm distance from the laser illuminated window) where it was carefully shielded from IR radiation, while the second thermocouple was positioned near the back region of the cell. The data from the two thermocouples were fed into a temperature controller. With this setup, the temperature controlled incoming air provided sufficiently stable temperature conditions, although the actual temperature inside the cell could not be determined. The temperature was measured on the surface of the SEOP cell at the thermocouple locations during ramping and steady-state processes. Typical temperature difference across the cell was less than 10 K after the steady–state conditions were reached.

### 2.4. Gas Mixtures

Research grade Xe (99.995% natural abundance, 26.4% ^129^Xe; Airgas, Rednor, PA, USA), Kr (99.995% natural abundance, 11.5% ^83^Kr; Airgas, Rednor, PA, USA), and N_2_ (99.999% pure, Air Liquide, Coleshill, UK) were used to prepare the gas mixtures used in this study. The mixtures with varying noble gas contents were prepared prior to the SEOP experiments using a custom built gas mixing system. The ‘standard mixture’ described in *section 2.6* required the use of research grade He (99.999% pure, Air Liquide, Coleshill, UK) in addition to other gases.

### 2.5. Determination of Obtained Polarization Values

For the determination of the actual polarization value, the integrated signal intensities of the hp noble gases were compared to the integrated signal intensity of a thermally polarized sample of the respective gas. For the thermal ^83^Kr NMR measurement, a 15 mm borosilicate sample tube was pressurized to 560 kPa of natural abundance Kr gas leading to 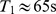 at 298 K [47]. Data were averaged from 360 acquisitions with a 360 s recycle delay time between pulses. Similarly, for the ^129^Xe thermal measurement, a sample tube was pressurized to 500 kPa containing 4 amagat of natural abundance Xe gas and approximately 1 amagat of O_2_ in order to reduce the longitudinal relaxation time to 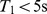 (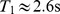 at 4.7 T [48]). Data were averaged from 120 acquisitions with 120 s recycle delay time between pulses. Taking into account the differences in concentration, pressure, and number of scans the integrated intensities from the thermal samples were compared with the integrated intensity of the hp samples to obtain the polarization enhancement over the thermal spin polarization.

For nuclei with arbitrary spin *I* the spin polarization *P* in a thermal equilibrium is given [45]:
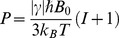
(1)with 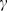 as the gyromagnetic ratio, 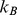 as the Boltzmann constant, and 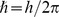
as the Planck constant. Eq. 1 assumes Boltzmann population distribution at high temperatures where 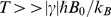, a condition that is fulfilled for the thermally polarized samples described above. Note that the thermal samples and the ‘standard mixture’ (described in *section 2.6*) where rerecorded with another NMR system (Bruker, Avance III at 9.4 T) in order to confirm the obtained hyperpolarization values with the Kea 2 spectrometer.

### 2.6. Accuracy of Polarization Measurements

The SEOP generated polarization can be measured with high precision through high field NMR spectroscopy. However, the polarization values will scatter due to fluctuations in the SEOP cell. For example, the cell surface will ‘cure’ after reloading with rubidium metal, probably due to redistribution of surface condensed Rb, and the obtained hyperpolarization will increase initially for up to a few hours for cells newly loaded with rubidium. Further, contamination with oxygen, CO_2_, or H_2_O will lead to a slow decrease in the obtainable hyperpolarization. Some of the cells that appear to be nearly identical lead to slightly different hyperpolarization values. Because of the many factors that may influence these measurements data sampling was randomized during parts of the experiment. To characterize experimental variation in cell performance over time a polarization value was obtained for a standard mixture (5% Xe, 5% N_2_, 90% He at 230±20 kPa and 373 K). This polarization value, averaged over a few experiments, was measured to be 44.0±5.4% and was further used for the ‘quality control’ test of a given SEOP cell. Three different SEOP cells that consistently achieved polarization values in this range were used during the course of the experiments. If the achievable polarization of a cell fell outside this range, it was cleaned and refilled with rubidium. Errors reported for the polarization measurements are based on the ±5.4% error of the standard mixture and scaled accordingly.

### 2.7. Data Analysis

Data analysis was performed using Igor Pro Version 6.2 from Wavemetrics (Lake Oswego, OR, USA). Fitting parameters for spin-exchange optical pumping were extracted using built-in non-linear least squares fitting algorithms.

### Background to the ^83^Kr and ^129^Xe SEOP Experiments

The unit ‘amagat’ for the number density [M_i_] of gas phase atoms or molecules is often used for convenience. In this work an amagat is defined as the density of an ideal gas at standard pressure and temperature of 101.325 kPa and 273.15 K and therefore 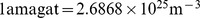. Note that the amagat was historically defined as the density of the specific gas at standard pressure and temperature resulting to the slightly different value for instance for xenon with 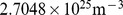 [49]. The small difference of less than 1% between the two definitions indicates almost ideal gas behavior for xenon at this condition.

### 3.1. Expected Pressure Dependence

The increase of the noble gas spin polarization as a function of the total pressure decrease is expected from [21], [50]:
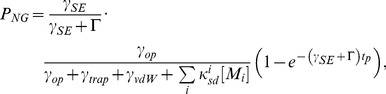
(2)where 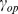 is the optical pumping rate caused by laser irradiation of the alkali metal atoms (i.e. by irradiation of rubidium (Rb) atoms with circular polarized light at the D_1_ transition at 794.7 nm for all experiments described in this work). In principle, the rate 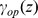 is a function of position within the pump cell due to the weakening of the laser in the optically thick medium [39], [51], however for the purpose of this work an averaged value 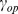 is assumed for simplicity, noting also the presence of significant gas convection in the SEOP cell [52]. The rate constant 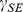 describes the spin exchange rate and 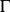 is the longitudinal relaxation rate of the noble gas atoms. The polarization, *P*_NG_, increases with increasing SEOP time, *t*_p_, until the contribution from the exponential term in Eq. 2 becomes negligible and the steady state value of polarization *P*_NG_ has been reached. The Rb electron spin polarization 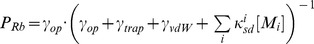 is limited by spin depolarizing collisions with inert gas atoms described by the gas (M_i_) specific rate constants 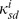 multiplied by the number density of the corresponding gas, 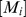. A further limitation is through radiation trapping described by the rate constant 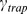 [30] that is further discussed below (see *section 3.3*) and by the rate constant 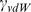 that is caused by spin rotation interactions (i.e. interaction of the Rb 5s electron spin with Rb-M_i_ molecular rotation - see *section 3.4*). A major contribution to the Rb depolarization in the gas phase at SEOP pressures 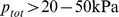 is caused by binary atomic collision. The rate constants caused by these interactions are directly dependent on the density of the respective atoms [33]. The rate constant of xenon is 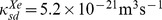 and is about 500 times larger than that of molecular nitrogen and more than 3 orders of magnitude larger than that of helium (see Table 1). Similarly, the rate constant of krypton, 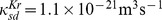, is a factor of 100 higher than that of molecular nitrogen. Therefore, even in the 95% nitrogen and 5% krypton gas mixture the contribution of molecular nitrogen to the overall Rb electron spin relaxation is only about 14% of the total gas phase relaxation caused by binary collisions. Moreover, in all other mixtures used in this work the nitrogen contribution to rubidium 5s electron spin depolarization through binary collisions is assumed to be below 4%.

**Table 1:** ^83^Kr and ^129^Xe literature rate constants used in Eq. 2.

Collision pair	Rb spin depolarization rate constants 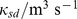	Spin exchange rate of van der Waals complexes^C^ 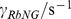	Binary spin exchange: 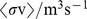	Characteristic pressure 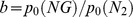
Rb-Xe	5.2×10^−21 ^ A	≈9.7×10^3 ^ D≈2.8×10^3 ^ E	1.0×10^−21 ^ D2.2×10^−22 ^ G3.7×10^−22 ^ E	
Rb-Kr	≈1.1×10^−21 ^ A	≈6.0^F^	2.1×10^−24 ^ F	
Rb-N_2_	≈9.4×10^−24 ^ B			
Rb-He	≈2.3×10^−24 ^ B			
Rb-Rb	≈8.1×10^−19 ^ A			
Xe-N_2_				0.275^E^
Kr-N_2_				1.90^F^

A From ref. [33] measured at 300 K.

B From ref. [51].

C Using 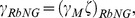
where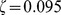 (assuming *P*_Rb_ close to 100%).

D From ref. [61].

E From ref. [27], values from this reference were used in calculations where multiple values have been reported.

F At 363 K from ref. [18].

G From ref. [60] for T = 373 K and B_0_ = 0 T.

### 3.2. Contribution of Rb-Rb Collisions

Unlike typical experiments at high SEOP pressure, depolarization of the rubidium electron spin due to rubidium-rubidium atom collisions may contribute significantly to Rb depolarization in the gas phase at low SEOP gas densities. The fairly large corresponding rate constant 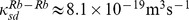
indicates that electron magnetic dipole – dipole interactions are responsible for the relaxation mechanism [51]. Depolarization due to Rb-Rb collisions depends on the rubidium number density [Rb] and is therefore a function of the SEOP cell temperature. An empirical equation (replacing an older, similar equation by Killian [53]) for [Rb] in m^−3^ as a function of temperature T in Kelvin is [54], [55]:
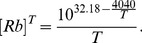
(3)


Using Eq. 3 one obtains that 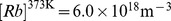 at 373 K. However Eq. 3 should be used with caution for Rb concentration calculations as uncertainties arise from the difficulty of proper temperature monitoring inside the SEOP cell during on-resonance irradiation with a high-powered laser as explained further in the text (see *section 4.3* for discussion of the correction factor, *c^Rb^*, to [Rb]).

The potential uncertainty in temperature is quite inconsequential for the rubidium depolarization in ^129^Xe SEOP since the rubidium density at a temperature of 373 K leads to a relaxation rate of 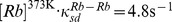 that contributes less than 2% to the Rb gas phase relaxation at the lowest pressure (5 kPa) and the lowest xenon concentration (5.0%) used. The significance of Rb-Rb collisions to the Rb depolarization decreases further as the total gas pressure and the xenon concentration increase. However, the situation is quite different in ^83^Kr SEOP. Firstly, the rate constant 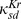 is about 5 times smaller than 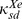, thus increasing the relative importance of 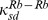 for the rubidium depolarization. Secondly, ^83^Kr SEOP produces the highest nuclear spin polarization at 433 K and, according to Eq. 3, 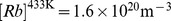. This translates into 27 fold increase in Rb concentration as compared to 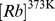 and Rb-Rb collisions contribute therefore significantly to the rubidium depolarization, in particular at low SEOP pressures. For example, at 30 kPa total gas pressure the contribution of 
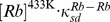
 to the Rb gas phase depolarization ranges from approximately 2% (for the 74% krypton mixture) to 5% (for the 25% krypton mixture) to about 20% for the leanest (5%) krypton mixture. Therefore uncertainties in SEOP temperature (and therefore [Rb]) can affect the second term in Eq. 2 for ^83^Kr SEOP.

### 3.3. Radiation Trapping

Molecular nitrogen is an important component of an SEOP gas mixture because it can, unlike mono-atomic noble gasses, dissipate energy from excited rubidium electronic states into vibrational modes [32], [56]. This non-radiative relaxation pathway reduces rubidium fluorescence, depending on the N_2_ number density [30]. In SEOP mixtures with high rubidium density [Rb], fluorescence may be detrimental to the Rb spin polarization because it can lead to radiation trapping where a single incident circularly polarized photon gives rise to multiple scattered photons that are arbitrarily polarized. Wagshul and Chupp [56] have reported a formula based on earlier experimental work [57] that quantifies the extent of quenching through N_2_. A slight modification of this formula, i.e. multiplication with the 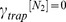 term from SEOP in the absence N_2_, leads to an expression similar to the one reported by Brunner and co-workers [52]:
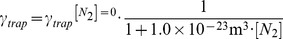
(4)where 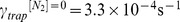 was obtained in an earlier ^129^Xe SEOP measurement [30]. Unfortunately, the effect of laser power, cell temperature, [Rb], and cell geometry on 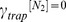 are little explored to date. For this work 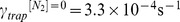 is assumed to provide a good approximation for ^129^Xe SEOP at 373 K but 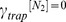 is expected to be significantly higher for ^83^Kr SEOP at 433 K due to the strongly increased rubidium density. Radiation trapping can be important at low pressure SEOP and is therefore included in Eq. 2.

### 3.4. Rb Depolarization Caused by Spin-rotation Interactions

At lower pressures with correspondingly longer lifetimes of the Rb-Xe van der Waals complexes, a significant Rb polarization loss is induced by spin rotation interaction [58]. In Eq. 2 this effect is represented by the rate 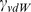. The functional dependence of 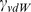 on SEOP gas pressure and composition is difficult to quantify. For an SEOP gas mixture with fixed concentration in the long-lifetime pressure regime (i.e. at very low pressures), the relaxation rate 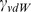 will increase with the pressure increase due to the intensified complex formation. At sufficiently high pressure the short molecular lifetime regime is reached and the further increase of complex formation with increasing pressure will be offset by higher breakup rates, thus resulting in pressure independent 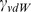. In this regime, the Rb nuclear-electron hyperfine interaction limits the influence of spin-rotation relaxation. At further pressure increase however, the very short lifetime regime is reached with a diminished hyperfine interaction and therefore, 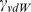
starts to increase again with increasing pressure until the hyperfine interaction has become completely negligible. For a 1% Xe, 1% N_2_, and 98% He SEOP mixture, a rate of 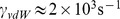 at 423 K (and an approximately 60% higher value at 353 K) has been reported for the short lifetime limit [58]. This value is comparable to that of 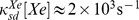 caused by binary collisions in ^129^Xe SEOP at 40 kPa and 373 K in the 5% Xe - 95% N_2_ mixture. The relaxation rate 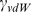 is however mixture dependent. For instance completely replacing helium by nitrogen should considerably reduce 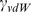 [59] as N_2_ facilitates the break-up of the Rb-NG van der Waals dimer better than helium. Unfortunately literature data of 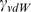 for the mixtures used in this work are not available. SEOP conditions in the current work are likely to create short to very short lived Rb-NG van der Waals complexes. Therefore, to a first approximation and within the scope of this work, 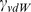 will be considered as pressure independent because of its general pressure independence in the short lifetime limit and because of its relatively small pressure dependence compared to binary relaxation, 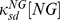 in the very short lifetime limit. In the lower pressure regime, where 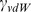 actually dominates Rb depolarization rate this crude approximation is destined to fail, therefore experimental data fitting with Eq. 2 (or modifications thereof) was not attempted in this pressure limit.

### 3.5. The Spin Exchange Rate

The spin exchange rate 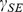 results from the added contributions of (1) spin exchange in rubidium - noble gas van der Waals complexes that is characterized by the rate constant, 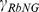 and (2) from spin exchange caused by binary collisions quantified by the velocity averaged binary spin-exchange cross section 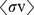. Literature values of 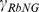 and 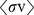 for ^83^Kr and ^129^Xe are listed in Table 1 [18], [27], [60], [61], while Eq. 5 shows the contribution of both rates to 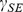 [27]:
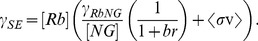
(5)


The rates 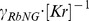 and 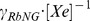 are comparable to their corresponding 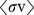 rates at a densities of 0.25 amagat and 0.4 amagat respectively (in the absence of nitrogen). In this density range, van der Waals dimers (mediated through three-body collisions) and binary collisions contribute about equally to the spin exchange. However, binary collisions will eventually dominate in the spin exchange process as the contributions from van der Waals complexes is expected to decline with the increase of the noble gas concentration and therefore its density [NG].

The N_2_ molecules in the SEOP mixture also contribute to the Rb-NG dimer break up. This contribution is quantified by the characteristic pressure ratio 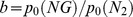 listed in Table 1 with the specific values for xenon and krypton [18], [27], [62]. The parameter *r* in Eq. 5 is the partial pressure ratio 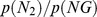 (or 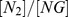 density ratio) in a mixture. The ratio *b* shows that a dilution of xenon in nitrogen can be beneficial to 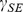. However, a dilution of krypton in nitrogen can be detrimental to 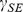 because the break up of van der Waals complexes is facilitated by nitrogen more than by krypton. Note however, that nitrogen is still beneficial for ^83^Kr SEOP because of its radiation quenching effect (*section 3.3*) and because 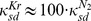 (*section 3.1*).

## Results and Discussion

### 4.1. Noble Gas Polarization as a Function of SEOP Gas Pressure

**Figure 2:**
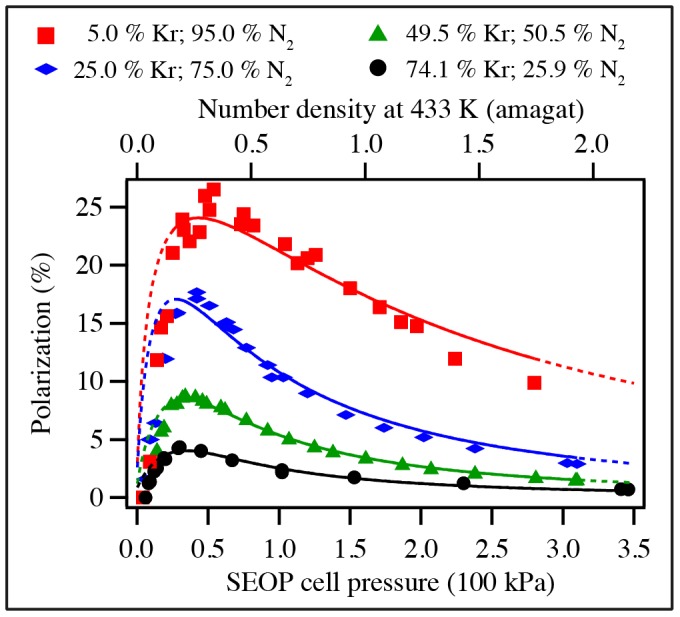
^83^Kr spin polarization, *P*, as a function of SEOP pressure. ^83^Kr spin polarization as a function of SEOP cell pressure and combined number density ([Kr]+[N_2_]) at 433 K for four different gas mixtures. See the legend in the figure for symbol explanation. Polarization data are detailed in Table 2. Data analysis using Eq. 8 with 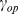 and 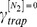 as fitting parameters is shown in solid lines and resulting values are reported in Table 4. Fitting of the data was also not attempted for values much lower than 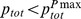; the dotted lines are extrapolations to pressure ranges outside the fitting region.

**Figure 3:**
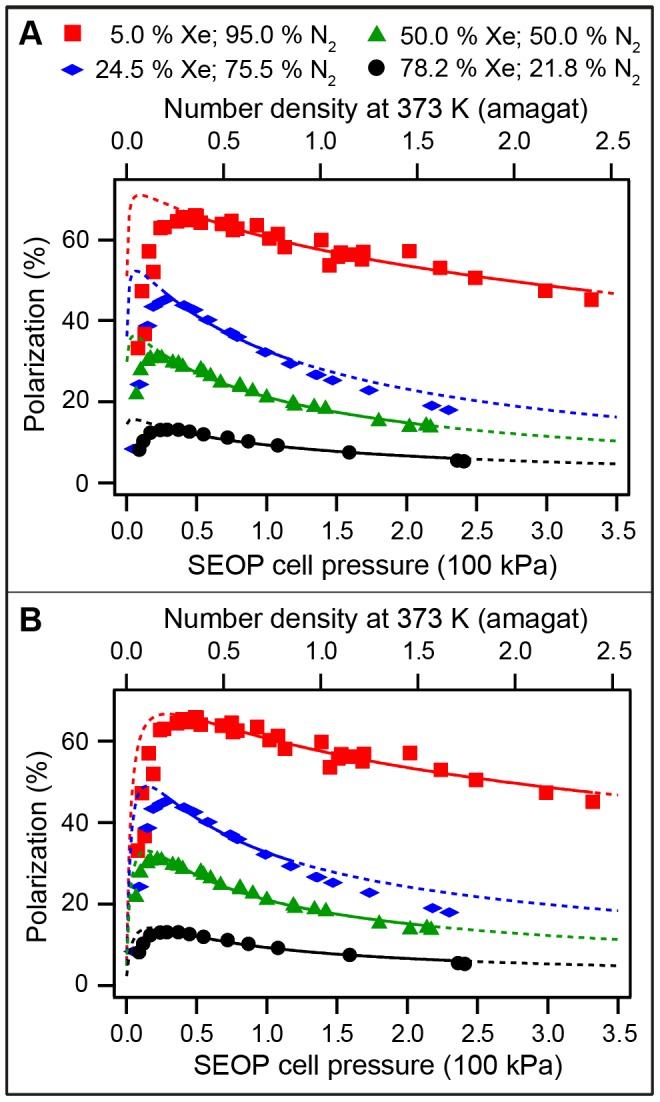
^129^Xe spin polarization, *P*, as a function of SEOP pressure. ^129^Xe spin polarization as a function of the SEOP cell pressure and combined number density ([Xe]+[N_2_]) at 373 K for four different gas mixtures. Please refer to the legend in the figure for symbol explanation. Polarization data are detailed in Table 2. **A**. Solid lines represent data analysis with Eq. 8. Extrapolation of these theoretical curves to pressure ranges outside the fitted region are shown by dotted lines. **B**. Same experimental data as in (A) but the solid lines represent now the data analysis using Eq. 8 with the pressure dependence of the Rb D_1_ absorption taken into account through Eq. 9. Extrapolation to pressure ranges outside the fitted region are shown by dotted lines. Fitting parameters for (A) and (B) are reported in Table 5A and 5B, respectively.

Steady state, or near steady state spin polarization was obtained for the ^129^Xe mixtures after about 6 min of SEOP at 373 K and a near steady state (approximately 80%) was reached after 8 min of SEOP for ^83^Kr mixtures at 433 K. The steady state polarization *P* is shown as a function of the total SEOP pressure 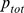 in Figs. 2 and 3 for hp ^83^Kr and hp ^129^Xe respectively. The noble gas polarization *P* of both isotopes in all mixtures increased as the total gas pressure was decreased from 350 kPa to below ambient in all studied mixtures. The maximum steady state polarization 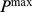 for hp ^83^Kr was obtained at a total gas pressure 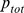, in the range of 35–50 kPa, depending on the krypton concentration used. Similarly, a polarization maximum was observed for hp ^129^Xe, however at a lower total pressure range of 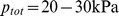. Reducing the pressure below these values resulted to a rapid drop in the steady state polarization of the noble gases. In order to facilitate the following discussions, the SEOP pressure that resulted to the highest observed steady state polarization 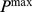, will be labeled as 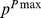. Table 2 lists 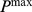
for various mixtures, the corresponding total SEOP pressure 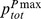, and the corresponding SEOP partial pressure 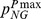.

**Table 2:** Maximum noble gas polarization 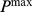, maximum apparent noble gas polarization 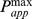, and corresponding gas pressures extracted from data of Figs. 2 and 3.

Mixture composition	Maximum polarization 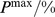	Apparent maximum polarization^A^ 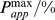	SEOP cell pressure and total gas density at maximum polarization 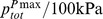 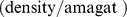		NG partial pressure and noble gas density, [NG], at maximum polarization 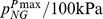 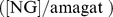		SEOP time and temperature
5.0 Kr;95.0 N_2_	**26.5±3.3**	***1.3±0.2***	0.54	(0.34)	0.03	(0.02)	8 minutes433 K
25.0 Kr;75.0 N_2_	**17.7±2.2**	***4.4±0.5***	0.42	(0.26)	0.11	(0.07)	
49.5 Kr;50.5 N_2_	**8.6±1.1**	***4.3±0.5***	0.41	(0.26)	0.20	(0.13)	
74.1 Kr;25.9 N_2_	**4.3±0.5**	***3.2±0.4***	0.30	(0.19)	0.22	(0.14)	
5.0 Xe;95.0 N_2_	**64.7±8.0**	***3.2±0.4***	0.46	(0.33)	0.02	(0.01)	6 minutes373 K
24.5 Xe;75.5 N_2_	**45.2±5.6**	***11.1±1.4***	0.28	(0.20)	0.07	(0.05)	
40.3 Xe;59.7 N_2_	**32.6±4.0**	***13.1±1.6***	0.22	(0.16)	0.09	(0.07)	
50.0 Xe;50.0 N_2_	**30.9±3.8**	***15.5±1.9***	0.22	(0.16)	0.11	(0.08)	
78.2 Xe;21.8 N_2_	**13.1±1.6**	***10.2±1.3***	0.37	(0.27)	0.29	(0.21)	

A
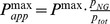
.

As can be seen from Table 2, the maximum ^83^Kr polarization of 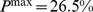 was reached for the 5% krypton - 95% nitrogen mixture at an SEOP pressure of 54 kPa. This is a remarkably high spin polarization for a quadrupolar spin system observed at ambient temperature. ^129^Xe SEOP at a pressure of 46 kPa using a 5% xenon mixture resulted to 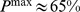
spin polarization. Both results were obtained with a 23.3 W laser irradiation that resulted in a power density of 2.6 W/cm^2^ at the SEOP cell front window.

Since hp noble gasses remain diluted without cryogenic separation process, the obtained polarization does not enable easy comparison with experiments that utilize cryogenic separation. It is therefore useful to define an apparent polarization, *P_app_*, scaled to the polarization, *P*, in the pure hp noble gas that would result to the same MRI signal.
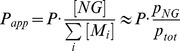
(6)


The apparent polarization, *P_app_*, provides a measure of the ‘usable’ spin polarization in MR experiments if the hp noble gas is not separated from the nitrogen after SEOP. Table 2 also lists the apparent maximum steady state polarization 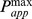. The highest 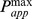 was obtained for krypton with the 25% and 50% krypton mixtures leading in both cases to 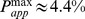. Mixtures with 40% and 50% of xenon lead to the highest values with 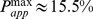. In cases where similar 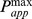
values are obtained for different SEOP mixtures, economical considerations will prefer the mixture with lower noble gas concentration, in particular when expensive isotopically enriched gases are used.

Note the maximum polarizations listed in Table 2 were generated every 6 minutes for hp ^129^Xe and every 8 minutes for hp ^83^Kr (and with slightly increasing values for *P*_Kr_ at SEOP times up to 18 min). The ideal pumping time for MRI applications however may be shorter than these values if polarization can be compromised in favor for faster experimental repetition.

### 4.2. SEOP Temperature

The three-body spin exchange rate 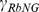 and the binary cross section 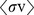 are both more than two orders of magnitude smaller for the Rb-^83^Kr system than for the Rb-^129^Xe system. The resulting small 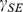 rate has two adverse consequences for ^83^Kr SEOP as predicted by Eq. 2. Firstly, a smaller 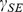 in the presence of a higher relaxation rate 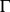 leads to a reduced steady state polarization *P* for ^83^Kr compared to that for ^129^Xe under otherwise identical SEOP conditions. Secondly, smaller 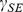 values further result in slower ^83^Kr SEOP polarization build up as compared to ^129^Xe SEOP, thus increasing the repetition time in MRI applications. In order to, at least partially, offset this effect [Rb] needs to be raised through elevated ^83^Kr SEOP temperatures. In addition to the increased [Rb], a further advantage of elevated ^83^Kr SEOP temperatures comes from reduced quadrupolar relaxation of ^83^Kr on the cell surface, as discussed in *Appendix 2* in Supporting Information S1. It was found that up to a temperature of 433 K the benefit from the increased spin exchange rate 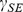 for ^83^Kr SEOP outweighs other detrimental effects arising from elevated temperatures. In contrast, a temperature of 373 K was found to produce the highest ^129^Xe spin polarization in this work. Examples of adverse effects at higher temperatures are increased Rb-Rb collision rates, as discussed in the *section 3.2,* and increased laser absorption in the rising optical density of the rubidium vapor phase.

### 4.3. Results from Inversion Recovery ^83^Kr SEOP Experiments

**Figure 4:**
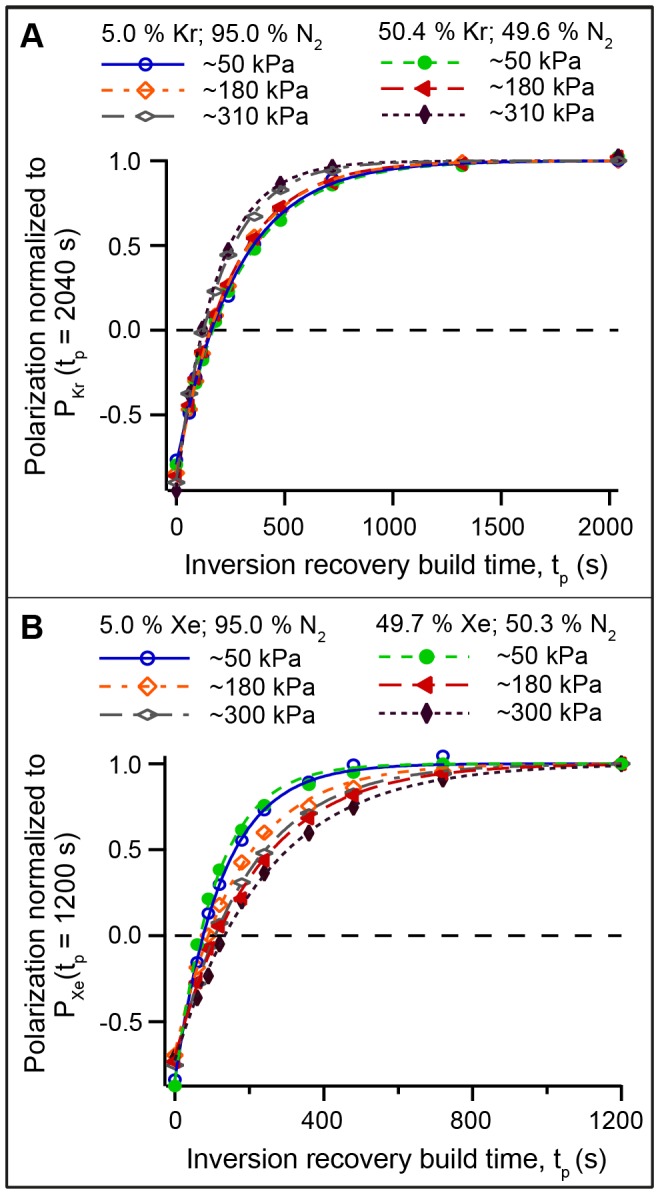
Inversion recovery ^83^Kr and^ 129^Xe SEOP. **A.** Inversion recovery of ^83^Kr polarization after SEOP time, *t*_p_, for two krypton-nitrogen gas mixtures at different SEOP pressures. Please refer to the legend in the figure for symbol explanation. **B.** Inversion recovery of ^129^Xe polarization after SEOP time, *t*_p_, for two xenon-nitrogen gas mixtures at different SEOP pressures. The inversion recovery data from both (A) and (B) were analyzed using Eq. S1. Polarization data were normalized to their values at 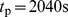 for ^83^Kr and 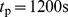 for ^129^Xe to visually compare the rate differences of the mixtures and pressures. The obtained rate constants from fitting of both (A) and (B) are reported in Table 3.

The noble gas self-relaxation rate 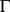 is difficult to obtain from published data as it is specific to some SEOP conditions, for example SEOP cell dimensions and its surface temperature. However, the combined rate constants 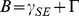 can be extracted from the time dependence of the polarization obtained in SEOP experiments according to Eq. S1 in *Appendix 1* in Supporting Information S1 (i.e. utilizing the time dependence of Eq. 2). In principal, build up curves can be measured directly inside the SEOP cell [28], [61], [63]. However, in this work the SEOP time dependence is determined through remotely detected NMR experiments (i.e. after hp gas transfer into the high field magnet) as no further experimental modification was required for the existing instrumentation. The drawback of this procedure was that the measurement of the build up curves required time-consuming point-by-point experiments. The data from inversion recovery ^83^Kr SEOP experiments (see *Appendix 1* in Supporting Information S1) are shown in Fig. 4A and the rate constants, 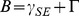, obtained from fitting with Eq. S1 are listed in Table 3.

**Table 3:** ^83^Kr and ^129^Xe values for 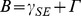 obtained from fitting of inversion recovery build up data (see Fig. 4) with Eq. S1.^A^.

Mixture	SEOP cell pressure (kPa)	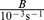	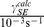	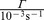 B
5.0% Kr; 95.0% N_2_	50	3.5±0.1	0.41	1.8±0.1
	180	3.9±0.1	0.36	2.5±0.1
	310	5.0±0.1	0.36	3.6±0.1
50.4% Kr; 49.6% N_2_	50	3.5±0.1	0.43	1.8±0.1
	180	4.0±0.1	0.37	2.5±0.1
	310	5.5±0.1	0.36	4.1±0.1
5.0% Xe; 95.0% N_2_	50	7.8±0.2	7.8	∼0
	180	6.0±0.2	3.7	1.2±0.2
	300	5.2±0.1	3.2	1.0±0.1
49.7% Xe; 50.3% N_2_	50	9.4±0.3	5.0	2.9±0.3
	180	4.7±0.2	3.0	0.8±0.2
	300	4.0±0.1	2.7	0.5±0.1

A The value of 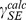 was calculated from Eq. 5 using literature values reported in Table 1. In the case of multiple literature values, ref. [27] values were used.

B Rubidium correction factors 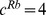 for ^83^Kr and 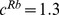 for ^129^Xe were used in the calculation of 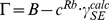.

The spin exchange rates 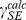 listed in Table 3 were calculated using Eq. 5 with the relevant literature values reported in Table 1. However, the experimental value 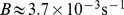 obtained from the inversion recovery experiments for ^83^Kr SEOP below 200 kPa presents a problem when combined with the calculated spin exchange rate values 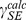 in order to determine the first fraction in Eq. 2, 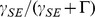. Using 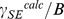, Eq. 2 predicts an upper limit for the ^83^Kr polarization of 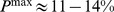. In reality, any experimentally measured value for *P*_Kr_ would be further reduced because of P_Rb_ <1 and due to incomplete (approximately 80%) build up at 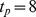 min in SEOP. In remarkable disagreement, the experimental data show polarization values of up to 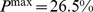 and 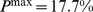 for the 5% krypton and 25% krypton mixtures, respectively (see Fig. 2 and Table 2).

The discrepancy between predicted maximum possible polarization and observed polarization may be due to incorrect literature data in Table 1 used for determining 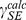. Note that the literature data was obtained at temperature conditions different from the ones used in this work. Another potential culprit is a wrong value of [Rb] obtained from Eq. 3 based on temperature measurements outside the cell. The temperature inside the cell under high power laser irradiation in the presence of the liquid rubidium metal is unknown. Wagshul and Chupp [51] noted a discrepancy of a factor of two or more in [Rb] under ^129^Xe SEOP conditions from the prediction by the equilibrium vapor equation. Further doubt about [Rb] determination through external temperature measurements arises from Raman spectroscopical experiments by Happer and co-workers that provide access to the *in situ* temperature distribution within the SEOP cell by measuring the rotational - vibrational N_2_ temperature [64]. The internal temperatures were found to substantially exceed those measured externally at the cell outside surface. Finally, a numerical simulation study [52] also draws a very complex picture about a non-uniform temperature distribution within a static SEOP cell with significantly elevated internal temperatures. The same, perhaps amplified problem may occur for ^83^Kr SEOP experiments that are run at the cell outside temperature of 433 K. A correction factor c^Rb^ for the rubidium concentration from Eq. 3 is therefore introduced for this work. It follows from the discrepancy between observed and calculated 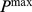 described above, that c^Rb^ >2. An upper limit for the correction factor c^Rb^ <8 is obtained from the fact that 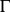 cannot be negative. Further, the upper limit can be reduced to c^Rb^ <6 if one assumes that relaxation rate 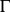 of ^83^Kr is not significantly lower than typical rates found for ^129^Xe under SEOP conditions. Further determination of c^Rb^ for ^83^Kr SEOP was not possible from the data in this work, however the qualitative outcome of the fittings in Fig. 2 is not strongly affected within the range 2< c^Rb^ <6. The correction factor was set to 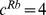
for further data analysis in Fig. 2.

The similarity in the 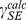 values in Table 3 for ^83^Kr SEOP is caused by the [Kr] independent rate constant 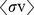 that dominates over the 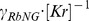
term even at the low pressures of 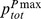 for all krypton mixtures. As pressure 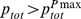, the van der Waals contributions will be even further marginalized. As a consequence, the inversion recovery ^83^Kr SEOP curves in Fig. 4A all display similar time dependence at SEOP pressures below 200 kPa. At 310 kPa, the combined rate constant is increased due to the increased relaxation rate constant 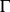. The functional form of the pressure dependence of 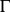 is explored in *Appendix 2* in Supporting Information S1. Rewriting Eq. S4 as a function of the krypton number density and using 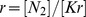
leads to:
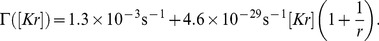
(7)


### 4.4. ^83^Kr Polarization vs. SEOP Pressure Dependence above 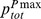


The pressure dependence of the ^83^Kr polarization, shown in Fig. 2, should be described in principle by Eq. 2 for SEOP pressures above 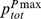. Most of the relevant parameter are listed either in Table 1 or described by Eqs. 3, 4, 5, and 7. The equation used for fitting of the data in Fig. 2 is:
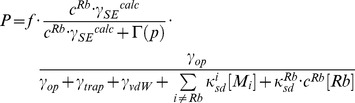
(8)where 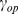 and 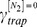 (in Eq. 4) were used as fitting parameters. The correction factor 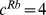 was used for [Rb], as described in *section 4.3*. A functional form of 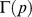 is given by Eq. 7 (also based on 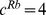). The scaling factor *f* = 0.8 in Eq. 8 accounts for the limited SEOP duration of 8 min that caused the polarization build up to be approximately 80% completed. The rubidium electron spin relaxation due to spin-rotation interaction in van der Waals complexes is represented by the rate 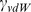 that is assumed to be constant under the SEOP conditions used in this work (see *section 3.4*). When used as a third fitting parameter, 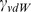 consistently emerged with negative or near zero values with little influence on the other fitting parameters, indicating small to negligible spin-rotation interactions for ^83^Kr. It was therefore set to zero and the results for 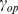
and 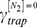 are listed in Table 4.

**Table 4:** Values for 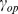 and 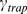 from fitting experimental data of ^83^Kr spin polarization as a function of SEOP cell pressure in Fig. 2 using Eq. 8.^A^.

Mixture	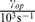	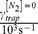
5.0% Kr; 95.0% N_2_	4.3±0.4	125±20
25.0% Kr; 75.0% N_2_	3.1±0.1	100±13
49.5% Kr; 50.5% N_2_	2.37±0.04	164±6
74.1% Kr; 25.9% N_2_	1.49±0.09	139±15

A A rubidium correction factor of 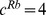 was used for the fittings of data in Fig. 2 with Eq. 8. The rate constant 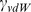 typically resulted to values close to zero but with large error values. Within its error margins 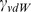 had little influence on the other fitting parameters and was set to 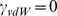 for the fittings reported in this table.

At a first glance, the fitting result in Fig. 2 (solid lines) appear to demonstrate that Eq. 8 qualitatively describes the dependence of the ^83^Kr SEOP polarization on [Kr] at pressures above 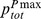. The obtained function describes the experimental observation reasonably well beyond the fitting range (see dashed line). The resulting 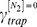 rate constants are fairly consistent but are about three fold increased compared to previously reported ^129^Xe SEOP data [30]. These values are quite high but an increase in 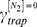 with increasing rubidium density is expected. The 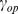
rates listed in Table 4 are low and indicate low pumping rates as it would be expected for an optically thick medium with high [Rb]. The 2.8 fold decrease of 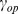 with increasing krypton concentration is further discussed in *section 4.8*.

### 4.5. Results from Inversion Recovery ^129^Xe SEOP Experiments

In contrast to ^83^Kr SEOP, the time behavior of the ^129^Xe SEOP polarization shown in Fig. 4B depends strongly on total pressure and gas composition (see Table 3). This observation is in agreement with previous work [28] and was expected since 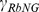, i.e. the van der Waals contribution to the spin exchange rate caused by three-body collisions, plays a more dominant role for ^129^Xe SEOP than for ^83^Kr SEOP. An increased 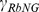 relative to the rate 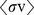 caused by two body collisions will result in a stronger noble gas density dependency for 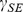 in Eq. 5. Furthermore, the time scale of the inversion recovery is accelerated at low xenon density compared to that of ^83^Kr (Fig. 4A). However, at high [Xe], 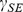 is reduced and the ^129^Xe SEOP time dependence (i.e. the rate constant 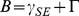) becomes similar to that of ^83^Kr SEOP at high [Kr]. The reason for the similar *B* values at high noble gas densities are of course different for the two isotopes: The dominating term in ^129^Xe SEOP is 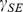 that decreases with [Xe], whereas 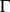 is assumed to be pressure independent. The ^83^Kr SEOP time dependence, on the other hand is controlled through 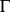 that increases with [Kr] while 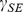 rate of ^83^Kr is mostly pressure independent.

The combined rate constants 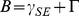 and the rates 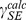 for ^129^Xe, as listed in Table 3, imply that the correction factor for [Rb], if needed at all, must be 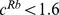 because of the requirement 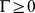. Once again, 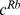 cannot be further determined and the average 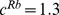 of the range is taken. Furthermore, the assumption is made that 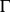
is caused mainly by interactions with the surface and is therefore pressure and gas composition independent. This seems to be indeed the case with the exception of the data taken at 50 kPa that scatter widely. However, for ^129^Xe SEOP at this pressure the values for 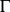 are relatively small compared to *B* and a significant error is not unlikely. Excluding 50 kPa data and averaging the 180 kPa and 300 kPa data one obtains 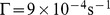
_ using_ 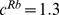. Note, for 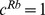 it follows that 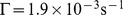 in better agreement with data by Goodson et al. [28] who previously determined 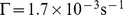 in a coated SEOP cell. However, as will be discussed in the following section, the exact value is not very important for the description of ^129^Xe SEOP in this work.

### 4.6. P_Xe_ vs. SEOP Pressure Dependence above 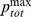


A qualitative analysis of the data shown in Fig. 3A was attempted with Eq. 8 derived from Eq. 2 with the inclusion of the correction factor for the rubidium density, 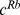. During the fitting procedure the rates 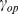 and 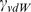 were used as the fitting parameter with the correction factor set to 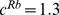 and the nuclear relaxation term to 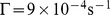. Unlike for ^83^Kr SEOP that is run at a temperature of 433 K, the radiation trapping term for ^129^Xe SEOP could be taken from literature data with 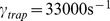 [30]. Furthermore, the SEOP duration was long enough to reach the steady state polarization value and therefore one could set *f* = 1. The rest of the constants used in the fitting procedure were taken from Table 1, in the case of the multiple choices of the literature data the constants from reference [27] were used. The resulting fits over the pressure range from 45 to 240 kPa are displayed (solid lines) in Fig. 3A (see also Table 5A for the relevant fitting parameters). The theoretical curves were further extended over the entire pressure range using the values for 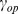 and 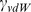 obtained from fitting (dotted lines). Although, fitting curves using Eq. 8 seem to qualitatively describe the experimental behavior in Fig. 3A, the results listed in Table 5A are not within the expected range. The optical pumping rate constant are quite high and, the rate constant 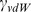 values are about one order of magnitude higher than a previous literature value for a 1% Xe, 1% N_2_, and 98% He SEOP mixture with 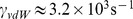 at 353 K [58] (see *section 3.4*). Furthermore, increasing [Xe] and decreasing [N_2_] should lead to increasing 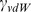, however the value for the mixture 78.2% Xe drops below 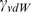 for all other mixtures and exhibits an unacceptably high error.

**Table 5:** Values for 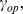 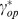, and 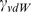 rates obtained from the fitting of experimental data of ^129^Xe spin polarization as a function of SEOP cell pressure (Fig. 3) using Eq. 8.^A^.

Mixture	A. Data fitting using Eq. 8 (Fig. 3A)		B. Data fitting using Eqs. 8 and 9 (Fig. 3B) 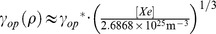	
	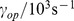	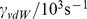	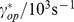	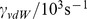
5.0% Xe; 95.0% N_2_	44±4	15±2	19.1±1.0	3.2±0.4
24.5% Xe; 75.5% N_2_	27±2	19±3	17.9±1.0	3.9±1.0
50.0% Xe; 50.0% N_2_	34±1	50±3	20.6±0.5	10.6±1.1
78.2% Xe; 21.8% N_2_	25±2	10±20	13.0±0.6	22±3

A Fittings of data in Fig. 3 using Eq. 8 used the following parameters: *f* = 1, 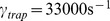,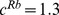 and 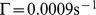.

Note that the general appearance of the overall shape of the fitting curves is not dramatically affected by 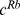
(at least within the range 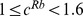), nor do the resulting values for the fitting parameters change significantly. Generally, the larger 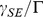 ratio makes the first term in Eq. 8 less important for ^129^Xe SEOP compared to ^83^Kr SEOP. However, the unsatisfactory results of the data fitting with Eq. 8 will need some further considerations. The rubidium D_1_ absorption linewidth may hold important information for the second term in Eq. 8 and may provide a better understanding of the experimental data. The effect of the D_1_ linewidth is discussed in the following section.

### 4.7. Non-linear Pressure Broadening of the Rb D_1_ Absorption Linewidth

**Figure 5:**
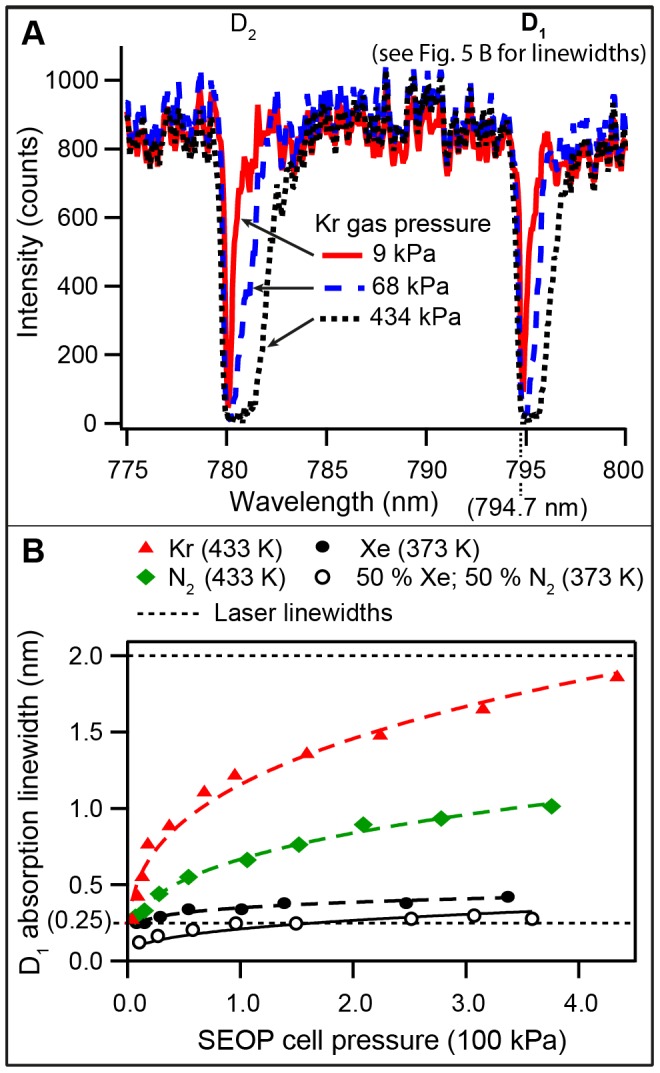
Rubidium IR absorption linewidth as a function of gas pressure. **A**. IR absorption spectrum of Rb in the SEOP cell containing pure krypton gas at 433 K at three different pressures as detailed in the figure legend. The absorption lines experience a pressure broadening and, to a lesser extent, a shift to higher wavelengths with increasing pressure. **B**. Rb D_1_ absorption linewidth as a function of SEOP cell pressure at 433 K for pure krypton (solid red triangles), for pure N_2_ at 433 K (solid green squares), for pure xenon at 373 K (solid black circles), and for a mixture of 50% xenon with 50% N_2_ (open black circles). The pressure dependence of the absorption linewidth can be approximately described by 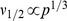 (dashed lines). Eq. 9 was concluded from the observed linewidth dependence. The linewidth of the narrowed laser and the broadband laser are 0.25 nm and 2.0 nm respectively, and are indicated in the figure by horizontal dotted lines.

Fig. 5A shows IR absorption spectra of rubidium within the SEOP cell when illuminated by an incandescent light source. Spectra were acquired at 433 K with pure krypton for three pressures: 9 kPa, 68 kPa and 434 kPa. Only the D_1_ transition (i.e. the 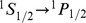
transition at 794.7 nm) and its linewidth are relevant for the SEOP studied in the present work. The pressure behavior of the D_1_ linewidth is depicted in Fig. 5B. Further theoretical analysis suggests that a [Xe]^1/3^, [Kr]^1/3^, and [N_2_]^1/3^ functional form provides a reasonably good description of the absorption linewidth behavior over the studied pressure range. The non-linear Rb D_1_ line dependence on gas density dependence is in contrast to the linear gas density dependence usually found for alkali metal D_1_ or D_2_ transitions (see for instance [46], [65]). The cause for this unexpected behavior was not further investigated and the exact functional description would benefit from refinement in future research.

Fig. 5B shows that the linewidth in the presence of either krypton or N_2_ at 433 K is much broader than that in the presence of xenon at 373 K. The Rb absorption linewidth with N_2_ at 373 K was too close the resolution limit of the optical spectrometer used (i.e. 0.04 nm). The data demonstrates that all krypton-nitrogen mixtures at 433 K should lead to a D_1_ broadening that is much larger than the laser linewidth (0.25 nm – dashed line in Fig. 5B) at all pressures above 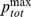.

However, a different situation occurs for xenon at 373 K, in particular in mixtures with N_2_. In these cases the laser linewidth may exceed the D_1_ linewidth and thus not all of the laser power will be absorbed. The effect of the linewidth is difficult to quantify, in particular since exact on-resonance irradiation can be disadvantageous as explored in detail by Wagshul and Chupp [51] and recently observed for high power irradiation by Wild and co-workers [66] and by Goodson and co-workers [67]. However, for this work the simple assumption is made that laser irradiation with a wider linewidth than the D_1_ linewidth will lead to a pressure dependent pumping rate that follows the same dependence as the D_1_ linewidth itself:
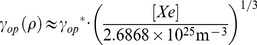
(9)with 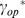 as the optical pumping rate at 1 amagat total gas density. The density dependent rate constant 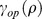 as defined in Eq. 9 replaces 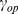 in Eq. 8. Using 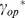 and 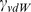 as fitting parameters with all other parameters kept identical to the ones used in *section 4.6*, fitting with Eq. 8 leads to the solid lines depicted in Fig. 3B with the values for rate constants listed in Table 5B. Once again, the theoretical curves were further extended over the entire pressure range using the values for 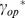 and 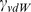 obtained from fitting (dotted lines). The results for 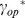 listed in Table 5B are similar to previous literature values [30] obtained under similar conditions and seem to be constant for different gas compositions except for the highest xenon concentration where a clear drop in 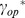 results. The value for 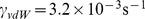 at 373 K for the mixture with 5% in Table 5 is identical to the literature value 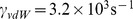
for a 1% Xe, 1% N_2_, and 98% He SEOP mixture at 353 K [58]. Further, with increasing [Xe] the values for 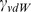 show a monotone increase. Overall, the consideration of the pressure dependence of the Rb D_1_ (Eq. 9) in Eq. 8 appears to result to more realistic values for 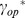 and 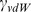. While there is little effect on the qualitative appearance between the fitted curves in Figs. 3A and 3B, the extended curve (dotted line) in Fig. 3B provides a better description of the observed data compared to the one in Fig. 3A.

It should be noted again that Eq. 9 should be handled with care since it is based on a number of simplifying assumptions. Firstly, neither the line shape of the pressure broadened Rb D_1_ transition nor the emission line shape of the frequency narrowed diode-array laser are Lorentzian or otherwise straightforwardly defined. Further, at high xenon concentration and pressure, the adsorption linewidth starts to exceed the laser linewidth causing the validity of the underlying concept in Eq. 9 to end. This may be the case in particular at high SEOP pressures for the mixture containing 78.2% xenon. Another factor, not considered here, is the pressure dependent shift of the D_1_ transition. For ^129^Xe SEOP at 373 K this shift is small with 0.13 nm over the used pressure range for pure xenon. Although the shift is larger at 433 K with 0.43 nm over the used pressure range for krypton (see Fig 5A) it is still small compared to the D_1_ line broadening. Despite the limitation of Eq. 9, requiring more refinement in future research, the current work suggests that the effect of pressure broadening needs to be considered for a correct description of variable pressure ^129^Xe SEOP with narrowed lasers.

### 4.8. Thermal Properties of SEOP Gases

The 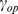 values for ^83^Kr SEOP listed in Table 4 of change by a factor of approximately 2.8 between the gas mixtures used. The 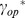
rates found in ^129^Xe SEOP summarized in Table 5B are less affected by [Xe] except for the mixture containing 78.2% xenon where the rate drops significantly. However, nothing in the general theory outlined in *section 3* gives rise to the expectation that 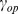 is affected by the noble gas-nitrogen ratio of the various mixtures. Nevertheless, at the same time it has been noted that the temperature gradient between the front and the back of the SEOP cell changed when SEOP mixture was altered.

The mixture dependent changes in the temperature gradient across the SEOP cell may have been induced by the different thermal conductivity of the used gas mixtures. Under the experimental SEOP conditions, N_2_ has an approximate 2.5 times larger thermal conductivity than krypton (and 4.5 times larger than xenon) [68]. Therefore, as the krypton or xenon concentration in the SEOP cell is increased, the decreasing thermal conductivity allows for higher temperature difference between the laser-illuminated front of the SEOP cell and its back. The consequences of this temperature gradient are unknown but changes in local rubidium concentration, thermal convection, and laser penetration are likely to lead to different convection patterns within the cell [52], [69]. Note also, that the heat capacity, 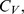 of N_2_ is more than 5/3 larger than that of a mono-atomic noble gas. Therefore, the corresponding changes between the gas mixtures may potentially have a profound impact on quantitative SEOP measurements and comparison of data between different noble gas mixtures needs to be handled with great caution. Due to the higher temperature, ^83^Kr SEOP may be stronger affected than ^129^Xe SEOP.

Thermal conductivity and heat capacity effects may explain the mixture dependent 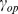 values but would of course also require mixture dependent 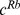 values. Unfortunately, the limited data in this work does not make the usage of a further fitting parameter reasonable in particular since the differences between the 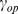 values are not too excessive.

However, a serious concern for the fitting of the experimental data would be SEOP gas pressure of the temperature, 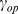, and 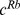. Fortunately, no effect on the pump cell temperature gradient with pressure changes has been noted. Moreover, the well-known equation for the thermal conductance, 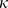, of an ideal gas is
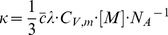
(10)where 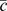 is the mean average velocity of the gas molecules, 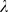 is the mean free path, 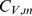 is the molar heat capacity at constant volume, [M] the density of the gas, and N_A_ is Avogadro’s number. The thermal conductivity of an ideal gas is pressure independent because the gas density is directly proportional to the pressure, whereas 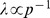 and 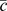 is also pressure independent.

### 4.9. Effect of Laser Power and Laser Linewidth

**Figure 6:**
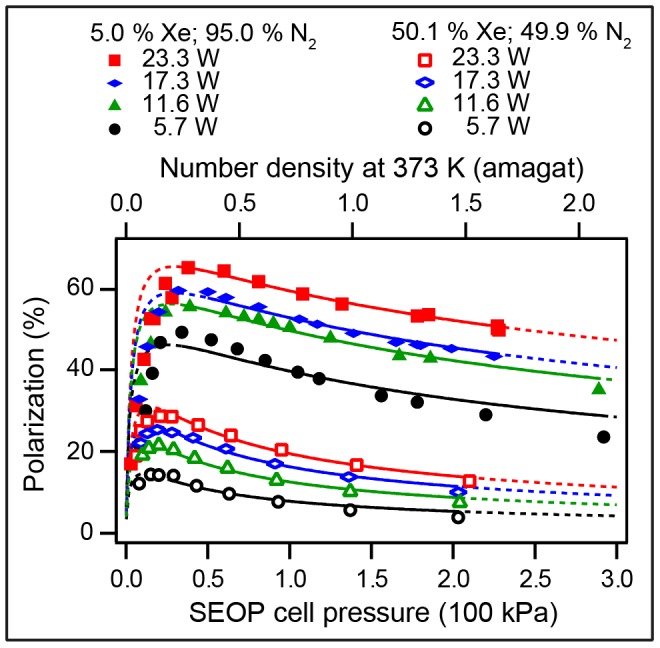
^129^Xe polarization, *P*, dependence on laser power. ^129^Xe spin polarization as a function of SEOP cell pressure for two different gas mixtures at four different SEOP laser power levels. Please refer to the figure legend for symbol explanation. The laser power was measured in the front of the SEOP cell. Data were analyzed using Eq. 8 (utilizing Eq. 9) within the fitting region (solid lines). Extrapolations to pressure ranges outside the fitted region are shown by dotted lines. The fitting procedure is discussed in *section 4.9* and the results of the data analysis are listed in Table 6.

The effects of laser power on the polarization curves are shown in Fig. 6. The power of the laser irradiation was adjusted in the linear polarized part of the laser beam rotating the 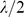 plate positioned in front of a beam splitter (see experimental section or Fig. 1B). This procedure allowed for the control of the laser irradiation power (incident at the SEOP cell) without changing the linewidth, the line shape, and irradiation pattern (i.e. beam shape). Fitting of the data was performed using Eq. 8 in the same fashion as in *section 4.7* using 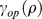 as defined in Eq. 9. The parameter 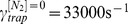 at 23.3 W power was taken from literature [30] and was scaled linearly with the relative decrease of laser power.

**Table 6:** Values of 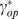 rates from fitting of the ^129^Xe spin polarization data for different laser powers and laser linewidths in Figs. 6 and 7 using Eq. 8.^A^.

Laser source	Mixture	Power (W)	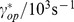
Narrowed laser(0.25 nm linewidth)	5.0% Xe; 95.0% N_2_	23.3	17.7±0.2
		17.3	12.8±0.2
		11.6	11.0±0.2
		5.7	6.9±0.3
	50.0% Xe; 50.0% N_2_	23.3	18.6±0.3
		17.3	14.8±0.3
		11.6	10.8±0.3
		5.7	6.3±0.3
Broadband laser(2 nm linewidth)	5.0% Xe; 95.0% N_2_	15.6	2.0±0.1
	50.3% Xe; 49.7% N_2_	15.6	1.6±0.1

A The rubidium correction factor was set to 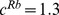. The values of 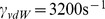 from Fig. 3B (Table 5B) for the 5% xenon mixture and 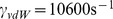
for the ∼ 50% Xe mixture were used. The parameter 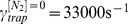 at 23 W power was taken from literature [30] and scaled linearly for all other powers with the relative decrease of laser power.

Measurements at 23.3 W power were performed redundantly under the same pumping conditions as the ones used for 5% and 50% Xe mixtures displayed Fig. 3. The resulting rates, 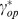, are listed in Table 6 for the two mixtures at various laser power levels.

The increase in 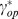 as the laser power is raised from 5.7 W to 23.3 W is 3.0 fold for the 50% mixture and is 2.6 fold for the 5% xenon mixture. However, the dependence of 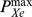
on laser power (see Fig. 6) is more pronounced for the 50% mixture (approximately 2.0 fold increase in the polarization 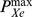 between 5.7 W to 23.3 W) compared to the 5% xenon gas mixture (1.3 fold increase). The increasing importance of laser power for SEOP with higher noble gas concentration is due to the second fraction in Eq. 8 that makes the 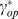 (or 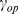) values more relevant for the obtained polarization, 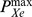, if the destructive rates 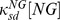 are high. Therefore higher laser power is particularly beneficial for higher noble gas concentration SEOP. This is an important observation for the concept of cryogen-free SEOP.

**Figure 7:**
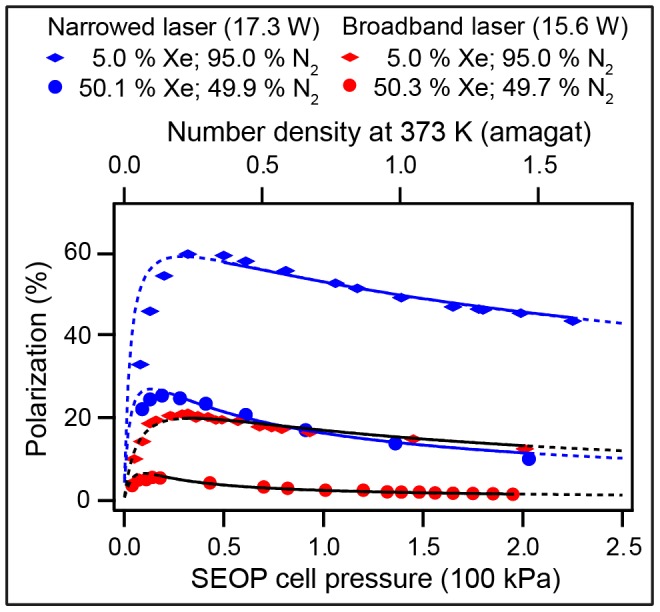
^129^Xe polarization, *P*, dependence on laser linewidth. ^129^Xe spin polarization as a function of SEOP cell pressure with the line narrowed (0.25 nm linewidth, 17.3 W) and FAP laser irradiation (2 nm linewidth, 15.6 W). Data were analyzed using Eqs. 8 and 9 for fitting region indicated by the solid lines as discussed in *section 4.9*. Extrapolation using the obtained values of the fitting coefficients to pressure ranges outside the fitting range are shown by dotted lines. Results of this data analysis are listed in Table 6.

Fig. 7 depicts a comparison of SEOP results obtained with a line narrowed (0.25 nm) Comet laser module using reduced laser power (17.3 W) and with a similar power (15.6 W) but using much larger linewidth (Coherent FAP, approximately 2 nm line width). Data were analyzed with Eq. 8 in identical fashion as above and the resulting 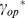 for broadband laser ^129^Xe SEOP are listed in Table 6. Clearly, laser line narrowing is beneficial for SEOP as it leads to a 9.3 fold increase of 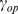 for the 50% xenon mixture and to the 6.4 fold increase for the 5% xenon mixture. Similar to the laser power trend, the resulting improvement of 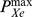 through line narrowing is particularly strong for SEOP with high xenon concentration. A 4.7 fold increase of 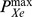 is observed in Fig. 7 for the 50% xenon mixture as compared to the 2.7 fold increase for the 5% xenon mixture.

### 4.10. Rapid Decrease of P_NG_ with Decreasing Pressure below 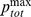


When the SEOP pressure was reduced below 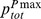 (i.e. 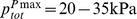 for ^129^Kr SEOP and 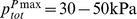 for ^83^Kr SEOP) a sharp decrease in polarization was observed. Note, that data fitting was limited to pressures above 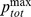, however simple extrapolation of the (high-pressure) fitting curves into the lower pressure region are shown as dotted lines in Figs. 2 and 3. These extensions seem to provide a remarkably good description of the low-pressure behavior. This result should however not be over-interpreted, in particular since the assumption of a constant 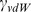 will fail in the low-pressure regions (see *section 3.4*). The rate 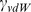, caused by spin-rotation interaction, will lead to significant depolarization at lower pressure but its effect is overestimated in this work because its absolute value will decrease with decreasing pressure.

There are further effects that contribute to the rapid polarization drop below 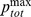. Radiation trapping, discussed in *section 3.3*, reduces the rubidium electron spin polarization. Radiation trapping will increase with lower 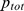 values, in particular in mixtures with high noble gas concentration (i.e. low N_2_ concentration) as described by Eq. 4.

A contribution to the polarization drop at pressures below 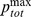, that is not accounted for in Eq. 8, may be caused by an optically dense boundary layer of rubidium at the cell window that is illuminated by the laser. This layer will reduce the resonant laser light penetrating the SEOP cell at any pressure. As demonstrated by Wagshul and Chupp [51] its effect is particularly detrimental at low pressures when the resonant absorption cross section of the rubidium is very high, leading to an almost complete absorption of the resonant laser light. The situation can be alleviated by detuning the laser to (slight) off-resonant illumination (not attempted in this work) and by the usage of very high laser power densities [51]. This effect was not investigated in this work.

Furthermore, the sudden drop in *P*_NG_ with decreasing SEOP pressure may be caused by a dramatic increase in rubidium relaxation due to the combination of increased diffusion and wall relaxation [33], [51]. The contribution of diffusion modes on the Rb relaxation in pure nitrogen becomes dominant and increases dramatically at pressures below 50 kPa of N_2_ [51], i.e. at a pressure slightly above 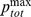 in the current work. This effect was also not further investigated in this work.

### Recompression of Low Pressure hp Noble Gases, Equivalent Flow Rates and Storage

This work demonstrated that SEOP with mixtures containing high noble gas concentrations can produce high spin polarization. This concept may be used as a pathway to hp noble gas MRI without the need for cryogenic separation. However, the drawback of this technique is that the hp noble gases need to be recompressed after SEOP. As shown previously by Imai et al. [36], diaphragm pumps can be utilized for low pressure ^129^Xe SEOP without significant depolarization. In the current work, recompression was found to maintain about 80% of the ^129^Xe polarization and approximately 60% of ^83^Kr polarization thus reducing 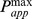 from 4.4% to approximately 2.6%.

Further development is needed to make recompression of larger volumes routinely available. The SEOP cell used in this work has approximately 75 cm^3^ volume and the ^129^Xe SEOP is complete every 6 min. Assuming 80% gas transfer, SEOP with the 50% xenon mixture at 22 kPa (see Table 2) leads to 1.8 cm^3^/min hp gas (at 298 K delivery temperature) with 12.4% apparent spin polarization, 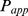. Similarly, SEOP with 25% krypton at 40 kPa results to an equivalent flow rate of 2 cm^3^/min hp gas with 2.6% apparent spin polarization.

The polarization and rates above have been obtained with a single 23.3 W laser (incident beam power at SEOP cell entry) and scaling of the volume should be possible by increasing laser power and SEOP cell volume. In any case the usage of multiple cells and lasers would increase the volume of hp gas per time unit. Furthermore, temporary storage of hp ^129^Xe at ambient temperature has previously been successfully demonstrated by Saam and co-workers [70] as a viable alternative to cryogenic storage. Further studies are required to explore temporary storage of hp ^83^Kr.

### Conclusions

Cryogen free production of hp ^83^Kr and hp ^129^Xe for practical MRI applications is possible through stopped flow SEOP with high noble gas concentrations at low total gas pressures. Without cryogenic separation the apparent polarization (as defined in Eq. 6) was 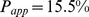 for hp ^129^Xe at a production rate of 1.8 cm^3^/min hp gas (volume at 298 K). Respectively, an apparent polarization of 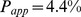 at a rate of 2 cm^3^/min was produced for hp ^83^Kr. These results were obtained using 23.3 W of laser power (incident at the SEOP cell) and a laser linewidth of 0.25 nm. Recompression of the hp gases after SEOP is a necessary step with this technique and preliminary work resulted to 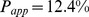 (for ^129^Xe) and 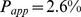 (for ^83^Kr) after recompression.

Current theory (Eq. 2) appears to provide a reasonable qualitative description of the SEOP gas pressure dependence of the polarization although several simplifications were used in this work. Overall, the practical application of current theory would benefit if more studies and published data were available. For instance, little is known about the actual spin-rotation parameter for various gas mixtures. Further, an experimental procedure to measure the temperature distribution within the SEOP cell would be very useful. In this work, a corrected value for the rubidium density [Rb] was used for ^83^Kr SEOP analysis (Eq. 8) that is 4 times higher than its predicted equilibrium value at the (externally) measured SEOP cell temperatures. A correction factor of 1.3 was used for ^129^Xe SEOP analysis, although correction proved to be less important compared to ^83^Kr SEOP. The rubidium density (and the pumping rate 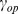 due to associated changes in laser penetration) also appeared to be dependent on the SEOP mixture, an effect attributed to different thermal conductivity of the various gas mixtures. Furthermore, the Rb D_1_ absorption linewidth dependence upon the SEOP gas pressure at 373 K was taken into account for the hp ^129^Xe data fitting (Eq. 9). The pressure dependence of the Rb D_1_ transition appeared not to be relevant for ^83^Kr SEOP because the D_1_ linewidth at 433 K is much wider than that of the narrowed diode array laser. However, a non-linear pressure broadening of the Rb D_1_ linewidth was observed in all cases and this unexpected behavior warrants further study.

High SEOP temperature is needed for ^83^Kr in order to increase the spin exchange rate 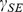 for ^83^Kr and to decrease the ^83^Kr relaxation rate 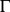. The results from ^83^Kr SEOP inversion recovery experiments suggest that surface relaxation is a strong contributor to 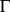 at SEOP below 200 kPa (see *Appendix 2* in Supporting Information S1 for discussions). Therefore, higher ^83^Kr spin polarization may be obtained through a reduction in surface to volume ratio using larger SEOP cells that reduce 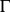 and thus increase the ratio 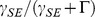 in Eq. 2.

The technique would benefit from future development focusing on practical gas recompression units, in particular for hp ^83^Kr, and on larger SEOP cell volumes to produce larger quantities of hp noble gas within a given time interval. Larger SEOP cells, that may also improve the polarization in ^83^Kr SEOP, will require increased laser power. Further increased laser power density at narrow laser line widths may be particularly advantageous for SEOP with high noble gas concentrations, as demonstrated in this work. Laser line narrowing to approximately 0.25 nm provides a crucial increase in ^129^Xe polarization compared to SEOP with a 2 nm laser and further narrowing would likely be helpful for ^129^Xe SEOP at low pressures. Finally, the general concepts of cryogen free hp noble gas production are by no means restricted to SEOP with rubidium. SEOP with cesium vapor [59], [71], [72] has recently been shown to increase the ^129^Xe polarization significantly compared to SEOP with rubidium [29]. The benefits of cesium vapor SEOP at low gas pressures, in particular with ^83^Kr, are still unexplored.

## Supporting Information

Figure S1. (TIF)

Supporting Information S1. (DOC)
