# Supplementary figures and images for: Pathway to Cryogen Free Production of Hyperpolarized Krypton-83 and Xenon-129
Source: PLoS One. 2012 Nov 27;7(11):e49927. doi: 10.1371/journal.pone.0049927 (PMC3507956; doi:10.1371/journal.pone.0049927)

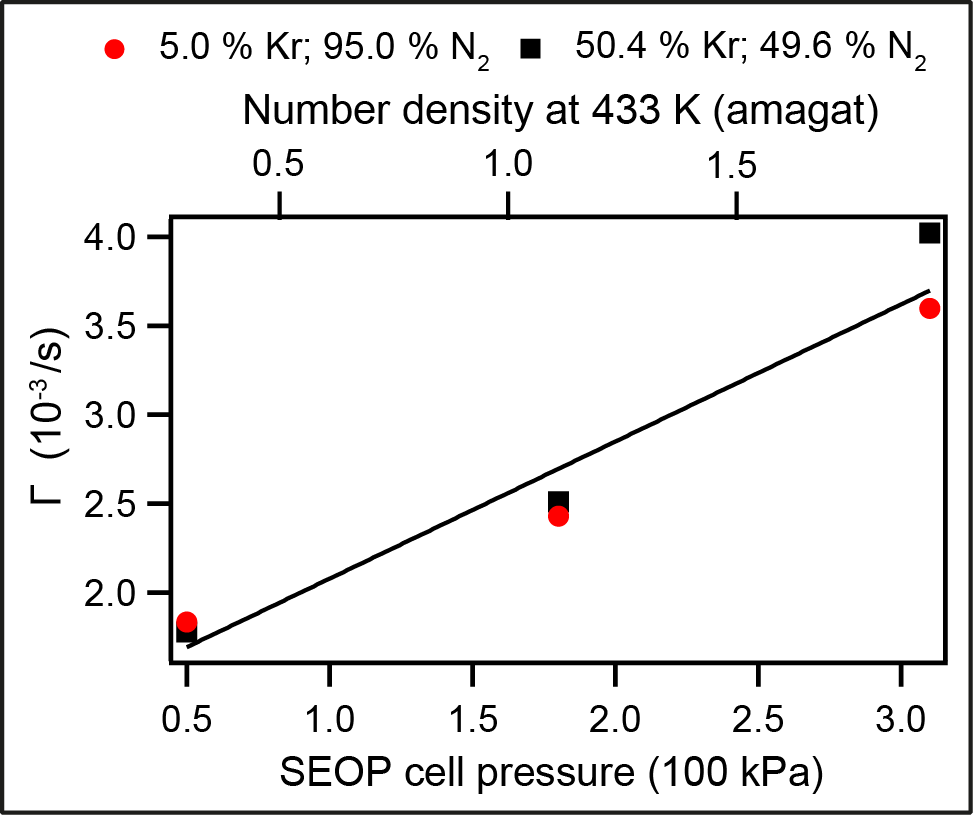

Supplement: Figure S1. — (TIF) [file pone.0049927.s001.tif]
