# Supplementary material for: Pathway to Cryogen Free Production of Hyperpolarized Krypton-83 and Xenon-129
Source: PLoS One. 2012 Nov 27;7(11):e49927. doi: 10.1371/journal.pone.0049927 (PMC3507956; doi:10.1371/journal.pone.0049927)
Supplement: Supporting Information S1. — (DOC) [file pone.0049927.s002.doc]

**Appendix S1. Inversion recovery SEOP**

The time dependence of the polarization, *P*_NG_, was probed in point by point experiments using ‘stopped flow’ SEOP for an incremented time period, $t_{p},$ followed by shuttling of the polarized gas mixture into the superconducting magnet for the high field detection. However, the rubidium concentration [Rb] will have to be regenerated after each shuttling event and the time dependence of this regenerative process needs to be eliminated from the data. Since pressure equalization is used for the shuttling of the hp gas, a further source of systematic error arises from incomplete gas transfer that leaves hyperpolarized gas in the SEOP cell and thus influences the outcome of the following experiment. The unwanted contributions of these two time dependent processes were previously avoided by maintaining SEOP like conditions for temperature, pressure, and illumination either in the absence of a magnetic field or by switching from circular polarized to linear polarized laser light during a 5 – 10 minutes recovery period. SEOP for the incremented *t_p_* duration is then started by either returning the magnetic field or by reinstating the circular polarization of the irradiation [1]. This type of experiment is reminiscent of ‘*saturation recovery*’ type of NMR relaxation measurements. An increased precision may be obtained in an ‘*inversion recovery*’ type of experiment where either the magnetic field is inverted or the circular polarization is reversed during a constant, precisely timed recovery period. In this work, circular polarized light was switched through rotation of the $\lambda/4$ plate instead of a faster magnetic field switch as SEOP was performed in the fringe field of the superconducting magnet that could not be altered straightforwardly. After returning the original circular polarization for the incremented time *t_p_* , the built up of *P*_NG_ was fitted to:

$P_{NG}\left(t_{p}\right)=C+A\left(1-2e^{-Bt_{p}}\right)$ (S1)

with fitting parameter $B=\gamma_{SE}+\Gamma$as the combined rate constant. The fitting parameters C normalizes the polarization $P_{NG}\left(t_{p}=\infty\right)\equiv1$. In the interest of time, the recovery (or ‘inversion’) period was kept to 12 min for ^83^Kr and 8 min for ^129^Xe, therefore the fitting parameter *A* was needed to account for incomplete inversion but also to correct for imperfections in the relative orientation of the $\lambda/4$ plate. Rotation of the $\lambda/4$ plate was accomplished manually, a process that took approximately 4-5 s. The switching time was acceptable for the slow build up rates in the experiments and automation of this process was not pursued.

**Appendix S2. ^83^Kr relaxation rate constant** $\Gamma$ **under SEOP conditions**

Fig. S1 shows the pressure dependence of the ^83^Kr relaxation rate constant, $\Gamma$, listed in Table 3 and displays the gas composition independence of $\Gamma$. The factors affecting the $\Gamma$ rates are worth further discussion since the surprisingly slow relaxation of this nuclear electric quadrupole isotope is of crucial importance for ^83^Kr SEOP. Note that the corresponding longitudinal relaxation times range between $T_{1}=244-555\text{ s}$ in this work.


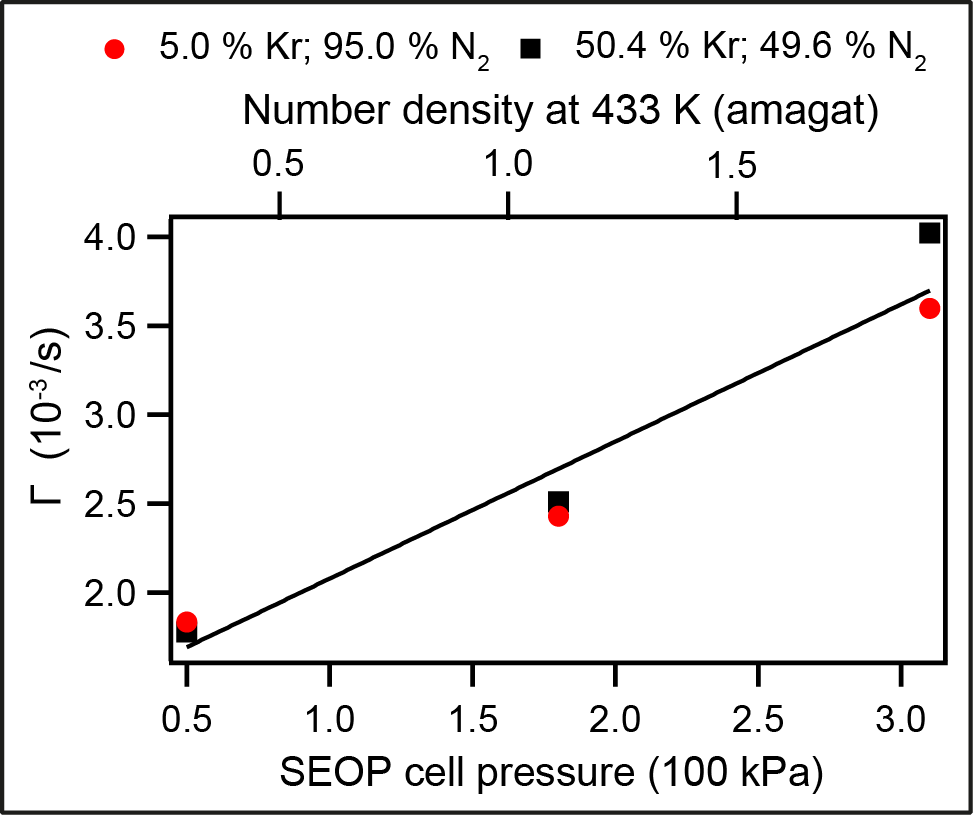


**Figure S1. ^83^Kr relaxation rate under SEOP conditions.** Dependence of $\Gamma$ on SEOP cell pressure for two different krypton mixtures as described in the legend. $\Gamma$ was calculated from the expression $\Gamma=B-c^{Rb}\cdot\gamma_{SE}^{calc};$ where $B$ was determined experimentally fitting Eq. S1 with the inversion recovery data of ^83^Kr in Fig. 4A. The value for $\gamma_{SE}$ was calculated from Eq. 5 using literature values displayed in Table 1, including the rubidium correction factor $c^{Rb}=4.$ The combined data from both mixtures was fitted assuming linear pressure dependence of $\Gamma$ (solid line) with the result summarized in Eq. S4.

Three events may contribute to the quadrupolar driven relaxation rate constant $\Gamma$; (i) binary collisions, (ii) Kr-Kr van der Waals formation, and (iii) surface adsorption:

$\Gamma=\frac{1}{T_{1}^{binary}}+\frac{1}{T_{1}^{vdW}}+\frac{1}{T_{1}^{surface}}$ (S2)

(i) The rate constant for relaxation caused by binary Kr-Kr or Kr-N_2_ gas phase collisions is [2]:

$\frac{1}{T_{1}^{binary}}=\left[\left(\frac{1}{T_{1}\rho_{Kr}}\right)_{Kr}+\left(\frac{1}{T_{1}\rho_{N_{2}}}\right)_{N_{2}}\cdot r\right]\rho_{Kr}$ (S3)

with $\rho_{Kr}$ and $\rho_{N_{2}}$ as the density of the respective gases in amagat and $r=\rho_{Kr}/\rho_{N_{2}}$. The density independent constants at ambient temperature are $\left(1/T_{1}\rho_{Kr}\right)_{Kr}^{298\text{ K}}\approx1.6\times10^{-3}\text{ amagat}^{-1}\text{s}^{-1}$ and $\left(1/T_{1}\rho_{N_{2}}\right)_{N_{2}}^{298\text{ K}}\approx2.3\times10^{-3}\text{ amagat}^{-1}\text{s}^{-1}$ [2]. Although the corresponding values for 433 K are not known, a linear dependence of $1/T_{1}^{binary}$on gas pressure (and therefore on $\rho_{Kr}$) can be assumed. Due to the similarity of the two rate constants, the resulting rate $1/T_{1}^{binary}$ is unlikely to be strongly affected by the gas composition. The limited amount of data shown Fig. S1 does not indicate a significant composition dependence of $\Gamma$. Linear fitting of the data leads to:

$\Gamma^{433\text{K}}\left(\rho_{tot}\right)=1.3\times10^{-3}\text{ s}^{-1}+1.2\cdot10^{-3}\text{ amagat}^{\text{-1}}\text{s}^{\text{-1}}\cdot\rho_{tot}$ (S4)

where the total gas density $\rho_{tot}=\rho_{Kr}+\rho_{N_{2}}$ was used making the simplifying assumption of an equal contribution from the two gases to the binary collision induced relaxation. The contribution $\left(1/T_{1}\rho_{tot}\right)_{Kr,N_{2}}^{433\text{ K}}\approx1.2\times10^{-3}\text{ amagat}^{-1}\text{s}^{-1}$ in Eq. S4 is in reasonable agreement with the literature data [2]. However, the interpolated zero pressure value $\Gamma\left(\rho_{tot}\to0\right)=1.3\cdot10^{-3}\text{ s}^{-1}$ cannot arise from binary gas phase collision. Two further relaxation mechanisms, described below, may contribute to this rate.

(ii) The rate constant for relaxation caused by ^83^Kr-Kr van der Waals dimers in the gas phase becomes independent of the gas density if the extreme narrowing condition is fulfilled [3]. This is the case for all experiments reported in this work. Further [4]:

$\frac{1}{T_{1}^{vdW}}\propto\frac{K}{\left(k_{Kr}+rk_{N_{2}}\right)}$ (S5)

where *K* is the equilibrium constant of the ^83^Kr-Kr complex formation and $k_{Kr}$ and $k_{N_{2}}$are the breakup rate constants due to collisions with krypton atoms and N_2_ molecules respectively. Eq. S5 and previous measurements at ambient temperature predict a decreasing relaxation rate $1/T_{1}^{vdW}$ with increasing nitrogen concentration [4]. Fig. S1 does not reflect such behavior and suggest therefore that $1/T_{1}^{vdW}$ does not contribute significantly under SEOP conditions at 433 K. A more thorough experimental study would be required to prove this since the small amount of data in Fig. S1 does not allow for a thorough analysis. However, a marginal contribution from van der Waals complexes to the overall relaxation is not completely unexpected due to the temperature dependence of the equilibrium constant *K* of dimer formation. At high temperature, the equilibrium will be shifted strongly towards the dissociated atoms. Therefore, van der Waals complexes are not the likely cause for the interpolated zero density $\Gamma$ rate in Eq. S4.

(iii) Another possible contribution to $\Gamma$ is caused by relaxation processes on the surface of the SEOP cell. The corresponding rate, $1/T_{1}^{surface}$, is independent of the gas composition (assuming no dramatic surface adsorption enthalpies differences between the gas components) and independent of gas pressure (assuming low surface coverage in the Henry isotherm limit). However, $1/T_{1}^{surface}$ has been demonstrated to depend on the surface to volume ratio [5] and is strongly accelerated by the treatment of model surfaces with silanizing agents [1,5,6,7]. In order to keep $\Gamma$ small, the SEOP cells used in this work have not been treated with silanizing or siliconizing agents. Increasing the temperature clearly helps with reducing the longitudinal ^83^Kr relaxation. Previously, a temperature dependent decrease of ^83^Kr longitudinal relaxation rates from $1/T_{1}=1.2\times10^{-2}\text{ s}^{-1}$ at 297 K to $1/T_{1}=4.5\times10^{-3}\text{ s}^{-1}$ at 433 K was demonstrated for a SEOP cell shaped container, similar to the one used in this work, but without laser irradiation and alkali metal present [8]. The 433 K value was obtained with a gas mixture of 95 % Kr and 5 % N_2_ at a density of approximately 1 amagat. Using $1.2\times10^{-3}\text{ amagat}^{\text{-1}}\text{s}^{\text{-1}}\cdot\rho_{tot}$ from Eq. S4, one finds a zero pressure intercept value of $\Gamma\left(\rho_{tot}\to0\right)=1/T_{1}^{surface}=3.3\times10^{-3}\text{ s}^{-1}$ and therefore a 2.5 fold faster surface relaxation than the one found in this work under SEOP conditions (i.e. laser irradiation and in the presence of rubidium metal). The reduced relaxation under SEOP conditions may be caused by a cell temperature that is significantly higher internally than the externally measured value. Alternatively, the presence of rubidium itself may reduce $1/T_{1}^{surface}$ through competitive co-adsorption [9]. In any case, $1/T_{1}^{surface}$ can be further reduced through the use of larger SEOP cells with decreased surface to volume ratios, thus leading to improved polarization values for ^83^Kr SEOP.

**References**

1. Stupic KF, Cleveland ZI, Pavlovskaya GE, Meersmann T (2011) Hyperpolarized Xe-131 NMR spectroscopy. Journal of Magnetic Resonance 208: 58-69.

2. Cleveland ZI, Meersmann T (2008) Binary-collision-induced longitudinal relaxation in gas-phase Kr-83. Journal of Chemical Physics 129: 244304.

3. Anger BC, Schrank G, Schoeck A, Butler KA, Solum MS, et al. (2008) Gas-phase spin relaxation of Xe-129. Physical Review A 78: 043406.

4. Cleveland ZI, Meersmann T (2008) Density-independent contributions to longitudinal relaxation in Kr-83. Chemphyschem 9: 1375-1379.

5. Stupic KF, Cleveland ZI, Pavlovskaya GE, Meersmann T (2006) Quadrupolar Relaxation of Hyperpolarized Krypton-83 as a Probe for Surfaces. Solid State Nuclear Magnetic Resonance 29: 79-84.

6. Wu Z, Schaefer S, Cates GD, Happer W (1988) Coherent interactions of polarized nuclear spins of gaseous atoms with container walls. Physical Review A 37: 1161-1175.

7. Raftery D, Long HW, Shykind D, Grandinetti PJ, Pines A (1994) Multiple-Pulse Nuclear-Magnetic-Resonance of Optically Pumped Xenon in a Low Magnetic-Field. Physical Review A 50: 567-574.

8. Cleveland ZI, Pavlovskaya GE, Stupic KF, LeNoir CF, Meersmann T (2006) Exploring hyperpolarized 83Kr by remotely detected NMR relaxometry. Journal of Chemical Physics 124: 044312.

9. Cleveland ZI, Stupic KF, Pavlovskaya GE, Repine JE, Wooten JB, et al. (2007) Hyperpolarized Kr-83 and Xe-129 NMR relaxation measurements of hydrated surfaces: Implications for materials science and pulmonary diagnostics. Journal of the American Chemical Society 129: 1784-1792.
